# An mHealth App to Support Caregivers in the Medical Management of Their Child With Cancer: Co-design and User Testing Study

**DOI:** 10.2196/33152

**Published:** 2022-03-16

**Authors:** Emily L Mueller, Anneli R Cochrane, Madison E Campbell, Sarah Nikkhah, Andrew D Miller

**Affiliations:** 1 Center for Pediatric and Adolescent Comparative Effectiveness Research Indiana University Indianapolis, IN United States; 2 Section of Pediatric Hematology Oncology Department of Pediatrics Indiana University Indianapolis, IN United States; 3 Indiana University-Purdue University Indianapolis Indianapolis, IN United States; 4 Human-Centered Computing Department, School of Informatics and Computing, Indiana University - Purdue University Indianapolis Indianapolis, IN United States

**Keywords:** child, adolescent, oncology, supportive care, mHealth, mobile health, cancer, pediatrics, children, digital health, health applications, parent, caregiver

## Abstract

**Background:**

Caregivers face new challenges and tasks when their child is diagnosed with cancer, which can be overwhelming. Mobile technology has the capacity to provide immediate support at their fingertips to aid in tracking symptoms, managing medication, and planning for emergencies.

**Objective:**

The objective of this study is to engage directly with end users and proxies to co-design and create a mobile technology app to support caregivers in the medical management of their child with cancer.

**Methods:**

We engaged directly with caregivers of children with cancer and pediatric oncology nurse coordinators (proxy end users) to co-design and create the prototype of the Cope 360 mobile health app. Alpha testing was accomplished by walking the users through a series of predetermined tasks that encompassed all aspects of the app including tracking symptoms, managing medications, and planning or practicing for a medical emergency that required seeking care in the emergency department. Evaluation was accomplished through recorded semistructured interviews and quantitative surveys to capture demographic information and measure the system usability score. Interviews were transcribed and analyzed iteratively using NVivo (version 12; QSR International).

**Results:**

This study included 8 caregivers (aged 33-50 years) of children with cancer, with most children receiving chemotherapy, and 6 nurse coordinators, with 3 (50%) of them having 11 to 20 years of nursing experience. The mean system usability score given by caregivers was 89.4 (95% CI 80-98.8). Results were grouped by app function assessed with focus on specific attributes that were well received and those that required refinement. The major issues requiring refinement included clarity in the medical information and terminology, improvement in design of tasks, tracking of symptoms including adjusting the look and feel of certain buttons, and changing the visual graph used to monitor symptoms to include date anchors.

**Conclusions:**

The Cope 360 app was well received by caregivers of children with cancer but requires further refinement for clarity and visual representation. After refinement, testing among caregivers in a real-world environment is needed to finalize the Cope 360 app before its implementation in a randomized controlled trial.

## Introduction

### Background

When a child is diagnosed with cancer, it is a life-altering event for both the patient and their caregivers [1]. After a new diagnosis, caregivers take on the immense burden of learning to navigate the health care system and provide at-home medical management. Although pediatric oncology providers play an important role in medical care, it is the caregivers who take on the burden of the hands-on, day-to-day care of the child with cancer. These roles of the caregiver can include the providing direct care, administering medication, assisting in activities of daily living, coordinating complex health care services, and providing emotional support [1-3]. Owing to the fact that many children with cancer had few serious medical needs before diagnosis, the weight of handling the new care demands can lead caregivers to experience distressful emotions, physical stress, and negative behavioral and physiological impacts [4-6]. A means by which we can improve caregiver outcomes could be to support their caregiving needs for their child with cancer.

Mobile health (mHealth), defined as the application of mobile or wireless communication technologies to health and health care [7], has tremendous potential to support caregivers in the medical management of their child with cancer. mHealth apps have been used successfully to support both patients and caregivers of adult patients with cancer [8-11]; however, none have been directly aimed at caregivers of children with cancer. In our recent investigation, we found that caregivers of children with cancer desired an mHealth app that would help them with the medical management of their child, specifically including medical knowledge, symptom tracking and management, and timely and convenient medication reminders [12]. These tracking and monitoring components of medical management could aid caregivers across the spectrum of their caregiving experience, including supporting them in the home setting, communicating with their oncology team about specific symptoms or concerns, and improving their preparedness when seeking urgent evaluation for a complication. In addition, preparing for potential medical emergencies is integral to caregiving for a child with cancer. Previous research has demonstrated that approximately half of the children with cancer will seek emergency department (ED) care within the first year after diagnosis [13]. Through our explorations of the experience of children with cancer and their caregivers when medical emergencies arose in the community setting, we found that the key components for emergency preparedness included the ability to easily connect with the oncology team, having a packing checklist, and an informational card to show the ED staff [14,15].

### Objectives

The objective of this study is to collaborate directly with key stakeholders, including caregivers of children with cancer and oncology providers, to place them at the center of the design and development process of an mHealth app to support caregivers in the medical management of their child with cancer. The hypothesis is that input from end users will lead to further and necessary refinements before implementing this app in a real-world setting.

## Methods

### Study Design

This is a pilot, mixed methods research study to engage directly with end users (ie, caregivers of children with cancer) and proxies (ie, nurse coordinators who triage sick calls) to co-design and create an app to support caregivers in the medical management of their child with cancer. There were two phases in this project: walking through prototyping of the app (phase 1), followed by alpha testing directly with caregivers (phase 2). First, we describe the intended functions of the mHealth app and its features, and then explain phases 1 and 2 of our study.

### Intended Functions of the mHealth App

#### Overview

**Figure 1:**
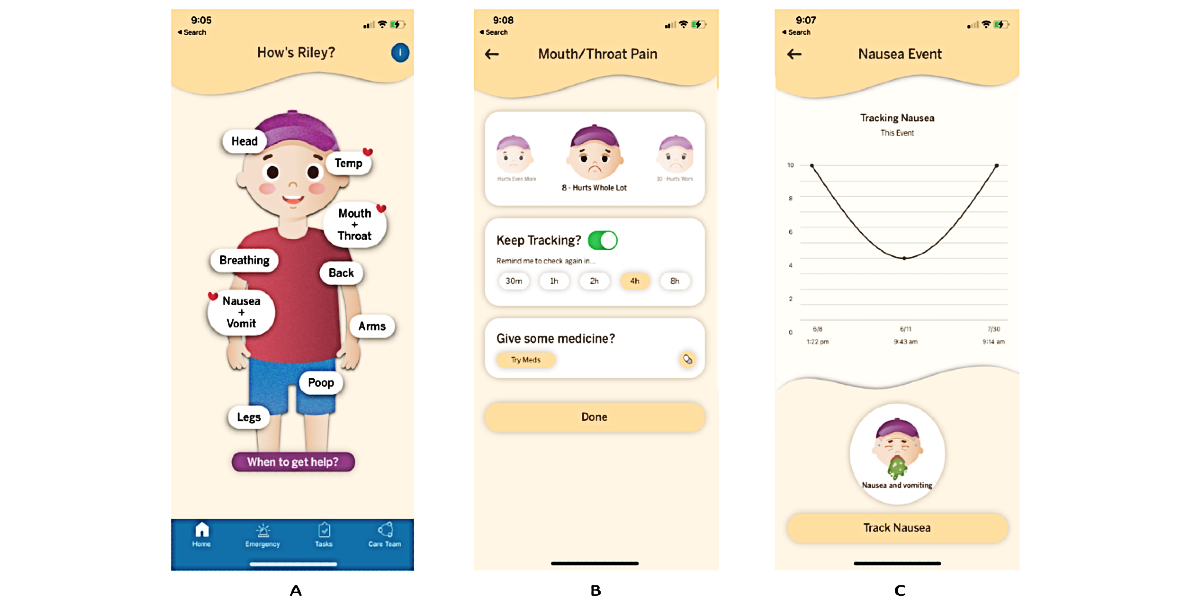
Images from the Cope 360 app including the (A) home screen view, (B) screen for documenting a symptom, and (C) screen for viewing the tracking of a symptom.

Our team strived to create an app that combined the features previously documented as desirable and functional for caregivers of children with cancer [12]. These desired features included medical management features such as medical knowledge, symptom tracking, and medication reminders [12]. Medical knowledge could consist of specific details about the child’s diagnosis, type of central line, and clinical recommendations for specific symptoms. The symptoms to track were based on literature related to the most common types of symptoms experienced by children with cancer, including pain, nausea and vomiting, diarrhea or constipation, fevers, and signs of breathing difficulties [16-20]. Medication reminders were created for both scheduled medications and supportive care medications if requested by the caregiver. It was also determined to be important to include a feature that aided caregivers in preparing to seek ED care for medical issues. The overall intent of the app was to assist caregivers in the medical management aspects of their child with cancer needs while they are in a home- or community-based setting. It was not intended to be used while patients were actively being evaluated by a medical professional or under the direct care of an oncologist (such as during hospital admissions for chemotherapy). Therefore, the app has three key functions: (1) patient information and caregiver team, (2) symptom tracking, and (3) emergency preparedness. Screenshots of the key screens are shown in Figure 1.

#### Patient Information and Caregiver Team

Patient information and caregiver team is where caregivers can add or view information on members of the caregiving team for their child. Under patient information and caregiver team, there is an open space to list the patient’s nickname; drop-down menus for listing the patient’s medical team based on our institution’s practices; and a toggle for if the patient has a central line, which then leads to a drop-down for line type. On the caregiver team screen, the primary caregiver can type their name, use a drop-down to characterize their relationship to the patient, determine which types of notifications they would like to receive, and upload a photo for their profile. On this screen, the primary caregiver can also invite other caregiver team members through a link to their phone contacts list.

#### Symptom Tracking

The purpose of symptom tracking is to assist caregivers in tracking common symptoms experienced by children with cancer, identified based on previous literature [16-20]. The symptom tracking feature is located on the home screen, where there is a cartoon representation of the patient that can be personalized by gender and 3 skin colors. There are bubbles for nine areas of symptom tracking, including head, temperature, mouth and throat, breathing, back, arms, nausea and vomit, poop, and legs. There is also a link to *When to get help?* on the home screen. Each symptom has an individualized tracking scale based on previously published or validated scales. We used the Faces Pain scale for head, back, arms, and legs [21]. The Baxter Retching Faces scale was used for tracking nausea and vomit [22] and the Bristol scale was used for monitoring poop [23]. The temperature tracking provides direct feedback based on the temperature input from the caregiver. The *When to get help?* screen includes reasons to call 911 with a direct link or reasons to speak with someone from the pediatric oncology team with a direct link to the clinic or after-hours services based on the day and time.

#### Medication Reminders

Either the oncologist or the nurse coordinator enters the patient’s current medications including scheduled medications and supportive care medications through the web-based application. Then, these are updated in the caregiver app, which will create reminders for scheduled medications. Once a symptom is tracked, the caregiver can also request reminders to administer supportive care medications until the symptom is no longer tracked.

#### Emergency Preparedness

The emergency preparedness plan screen allows the caregiver to create, practice, and enact a plan for seeking care for an urgent medical issue. The emergency preparedness plan screen will enable caregivers to pick their preferred ED and set up a contact plan with prescripted texts or a contact list to call and will provide a checklist of things to do, a packing list for items to bring, and finally, a *when you arrive* screen that can be shown to the ED staff.

### Phase 1: Development and Rapid Refinement With Proxy Users

On the basis of previously published research on prototyping an mHealth app for children’s oncology emergency planning, we learned that caregivers desire the ability to track symptoms and have medication reminders [12,15]. Therefore, we used these data to create the initial prototype, and then, we sought formative input from proxy users (ie, nurses) before initiating alpha testing with end users (ie, caregivers). We conducted rapid design interviews with nurse coordinators in our hospital system, who are health care professionals engaged in phone management and triaging of children with cancer who are experiencing medical emergencies in the home setting. At our institution, 7 nurse coordinators play this role. Demographic information, including age, gender, race, ethnicity, zip code, years of experience category, degree, and job role, was collected from the nurse participants. Nurse coordinator interviews were conducted using quick-and-dirty prototyping design methods by Buley [24], intended for proxy users of the final product. These interviews were conducted in person with the research team observing the nurse coordinator going through a series of tasks, including downloading the app, creating a profile, developing an emergency action plan, and opening and tracking each type of symptom (pain, nausea and vomiting, pooping, breathing, and fever). The nurse coordinators were observed for how often they encountered errors or if there was confusion with the intended function. They were encouraged to *think aloud* during the process, and the research team took notes [25,26]. The prototype was refined based on feasibility feedback provided by the nurse coordinators. These rapid prototyping sessions resulted in refinements to the app version in preparation for alpha testing with end users and proxies.

### Phase 2: Alpha Testing With End Users

In phase 2 of the project, we used alpha testing to refine the app with caregivers of children with cancer. First, demographic information from the caregivers was collected using a web-based survey, including relationship to the child with cancer, age, gender, race, ethnicity, zip code, marital status, annual household income, and education. Then, alpha testing was accomplished through an audio-recorded semistructured qualitative interview and a quantitative web-based survey. For the interview, participants were asked to perform a series of tasks to test the usability of the prototype using the same series of tasks as the nurse coordinators: downloading the app, creating a profile, developing an emergency action plan, and opening and tracking each type of symptom (pain, nausea and vomiting, pooping, breathing, and fever). They were encouraged to *think aloud* [26] and comment or ask questions as they moved through the app. Then, the interviewer would follow up to probe deeper into the comment or to obtain clarification. The interviews were audio and video recorded so that during analysis, the reviewer could see which screen was being referenced. At the completion of the interview, caregivers completed a web-based survey using the System Usability Scale (SUS) for the app [27,28].

### Collaboration and Ethics

Development and prototyping of the app were made possible through a partnership with Coactive Business Solutions of Indianapolis, Indiana. The Indiana University Institutional Review Board approved this study (number 1903250567).

### Study Population and Identification of Cases

A convenience sample of pediatric oncology nurse coordinators employed at Riley Hospital for Children was used for testing among health care providers in phase 1. Nurse coordinators were contacted via email and scheduled for an in-person interview during their typical workday. In phase 2, the participants were caregivers of a child with cancer (the child had to be aged <21 years), had adequate English language proficiency with grossly normal cognitive function, and had a child who was currently receiving cancer therapy at Riley Hospital for Children and at least 1 month had passed after initial diagnosis. Nurse coordinator interviews were conducted both in person and via Zoom videoconferencing. Caregivers were contacted by phone to schedule the interviews, which were conducted and recorded over Zoom videoconferencing owing to COVID-19 restrictions.

### Analyses

The research team created an initial codebook based on the series of tasks requested to be completed by each participant. For each task, codes were created for positive and negative comments. We conducted iterative thematic analysis on transcripts and notes from each interview. In each phase, interviews were conducted with participants (nurse coordinators in phase 1 and caregivers in phase 2) until no new information was gathered and thematic saturation was achieved [29,30]. Caregiver semistructured interviews were transcribed by a Health Insurance Portability and Accountability Act–compliant service and then analyzed using NVivo (version 12; QSR International) by three team members (MEC, ARC, and ELM). First, two team members (MEC and ARC) independently reviewed each transcript and assigned codes based on themes using an initial codebook based on the tasks that caregivers were asked to complete and comment on. Codes were revised based on new themes that emerged through data review [29,30]. A final review was performed with three team members (MEC, ARC, and ELM) until agreement on codes and themes was obtained. Findings from the transcripts were then grouped by similarity to create overarching themes. Data are presented as both features that worked well and those recommended for improvements in future versions of the app.

To evaluate usability, we chose to use the SUS [27,28], which has 10 questions on a 5-point Likert scale. The SUS has a calculated final score that is based on a well-established reference standard and is suitable for use even among small populations. A high SUS score indicates better product usability by the participants who evaluated it.

## Results

### Demographic Information of Phase 1 and Phase 2 Participants

A total of 6 nurse coordinators were interviewed in phase 1 of the prototype testing. Interviews lasted approximately 15 minutes on average. As presented in Table 1, all the nurse coordinators were women and White and non-Hispanic (6/6, 100%) and all of them had a Bachelor of Science in Nursing degree (6/6, 100%). Age ranged from 34-51 years, with a median age of 35 years. Job experience ranged from 3-5 years to ≥20 years and half of them (3/6, 50%) stated that they had 11-20 years of experience.

A total of 8 caregivers were interviewed for phase 2 of the prototype testing. Interviews lasted for approximately 25 minutes on average. As presented in Table 1, all caregivers were women, White, non-Hispanic, and parent-type caregivers (8/8, 100%). Half of the caregivers (4/8, 50%) had a child diagnosed with acute lymphoblastic leukemia, whereas the other half (4/8, 50%) had a child diagnosed with solid tumor type of cancer. Of the 8 children, 4 (50%) of them were undergoing chemotherapy only, whereas 3 (38%) were being treated with both chemotherapy and radiation and 1 (13%) was undergoing another type of treatment. Nearly all the caregivers were married (7/8, 88%), with 13% (1/8) of them being divorced. Most caregivers reported yearly household income of >US $75,000 and education level of college graduate or higher.

**Table 1:** Demographic characteristics of the participants alpha testing the Cope 360 app (N=14).

Characteristics		Caregivers (n=8)	Nurse coordinators (n=6)	
**Gender, n (%)**				
	Men	0 (0)	0 (0)	
	Women	8 (100)	6 (100)	
Age (years; n=7), median (range)		40 (33-50)	35 (34-51)	
**Race and ethnicity, n (%)**				
	White, non-Hispanic	8 (100)	6 (100)	
**Type of cancer, n (%)**				N/A^a^
	Acute lymphoblastic leukemia	4 (50)		
	Solid tumor	4 (50)		
**Type of therapy, n (%)**				N/A
	Chemotherapy only	4 (50)		
	Chemotherapy and radiation	3 (38)		
	Other	1 (13)		
**Type of caregiver, n (%)**				N/A
	Parent	8 (100)		
**Marital status, n (%)**				N/A
	Married	7 (88)		
	Divorced	1 (13)		
**Yearly household income (US $), n (%)**				N/A
	<25,000	0 (0)		
	25,000-49,999	1 (13)		
	50,000-74,999	1 (13)		
	75,000-99,999	2 (25)		
	100,000-150,000	1 (13)		
	>150,000	3 (38)		
**Education, n (%)**				
	Less than high school	0 (0)	0 (0)	
	High school or GED^b^	1 (13)	0 (0)	
	Some college	2 (25)	0 (0)	
	College graduate	3 (38)	6 (100)	
	Graduate degree	2 (25)	0 (0)	
**Job experience (years), n (%)**		N/A		
	0-2		0 (0)	
	3-5		1 (17)	
	5-10		1 (17)	
	11-20		3 (50)	
	≥20		1 (17)	

^a^N/A: not applicable.

^b^GED: General Educational Development.

### System Usability Score of Phase 2 Participants

When we evaluated the 8 caregivers’ SUS responses, we found a mean score of 89.4 (95% CI 80-98.8). This falls above the generally recognized lower limit of acceptability for technology applications (≥70) [27,28].

### Qualitative Exploration of Phase 2 (Caregivers) Interviews

For the qualitative evaluation of the caregivers’ experience with the app during alpha testing in the *lab-based* setting, responses were grouped by app function with common themes of either positive attributes or future areas for refinement presented. Representative quotes of caregivers are shown in Table 2.

**Table 2:** Key quotes from caregiver interviews, grouped by theme.

Theme and function			Key quotes
**App setup and planning**			
	App log-in and caregiver team creation	“This is nice, select from contacts. Okay, search. That part’s really nice.” “I know what a central line is because I’m a surgeon, but I’m not sure other people would know that.”	
	Emergency action planning	“Yeah. It just goes right to my contacts and pulled it through, so very easy. So, I added that.”	
	Task list and planning list	“Actually, I would probably add to this grabbing her medications only because the last time we went to the ER, we forgot them. Oh, my goodness sakes. We’ve been doing this for how long, and then we forgot it.” “I really like that because when you’re in that moment, it’s hard to remember everything...Yeah, that’s cool.” “This is helpful because I feel like I always forget something...”	
**Seeking emergency care**			
	When you arrive	“Yeah, and we have utilized two emergency room departments,...and both of them have been disastrous. So, just as I’m reading this, I’m like, oh my gosh. If I had something like this, I could be like, look, this is what has to happen. I think it would be huge.” “The first time we went to [a local hospital], the doctor was looking at me like, what do you want me to do?...So, I guess that would have been helpful to be able to show that to him like this is what their plan, what they recommend.”	
**Medical management of care**			
	Logging symptoms	“I think it’s a very convenient, very easy, very helpful because we may not write down as much as what we should, and this would be very easy to just pull up and push the buttons and say okay, this is what’s going on.” “Yeah. I mean, I think it would be better with words under it...”	
	Medications	“Right, but it’s cool though to have a listing of her medications. I don’t know what type of information...I think [it would be helpful to have] because I know we get the papers, clinic or an inpatient, but I know those hardcopies just get sort of lost in the shuffle.” “I would say having one app where you manage everything, including the regularly scheduled meds, which are really honestly extremely important. That’s the treatment.”	
	Miscellaneous	To-do list: “Yeah, and medicines would be good on there. Right now, I do it on a board, but it would be easier to do it in my phone, so I had it when I got over to the hospital.”	

### App Setup and Planning

#### App Log-in and Caregiver Team Creation

First, caregivers were asked to log in to the app using their phone number and were provided an access code through an SMS text message. All participants were able to successfully enter the app; however, 13% (1/8) of them had difficulty in receiving the access code but eventually received it. Next, they were asked to create an account. Almost all (7/8, 88%) of them commented that they were able to add other caregivers to the app with no or few difficulties. Of the 8 caregivers, 1 (13%) caregiver did not comment on whether they had any difficulties regarding this. A few caregivers (3/8, 38%) were initially confused if a port-a-cath (port) was considered a central line when adding patient information to create an account. However, once the central line was selected, they were able to select the port from a drop-down list of line types and understood the setup. Overall, caregivers were able to easily set up an account in the app with little to no assistance from the interviewer; however, not all of them commented on it.

#### Emergency Action Planning

Then, the caregivers were asked to set up an emergency action plan, which had several components. They were asked for their preferred ED, their contact plan (a place where they can set up SMS text messages or phone calls to other people if they are going to the ED); a *before you leave* section, where they can be reminded of tasks that need to be done before leaving for the ED; a packing list; a *when you arrive* section, which contained general instructions for the treatment of a child with cancer that can be shared with the ED; and finally, a *when to get help* informational section. Overall, the caregivers found it easy to set up an emergency action plan. Caregivers who added other people from their contact list to their emergency action plan said that it was easy to do. They also found it easy to edit SMS text messages when asked how they would do it by the interviewer.

#### Task List and Packing List

All caregivers appreciated the task list that included several prepopulated tasks including to call the oncology team and pack. There were additional tasks that caregivers could add to their task list including bringing home medications, packing the wheelchair, and seeking childcare for other children at home. When asked to add items to the packing list, many caregivers thought they had to press the plus button and then start typing instead of typing and then pressing the plus button. After they understood the correct method, they stated that it was easy to do. The caregivers had positive thoughts on the packing list with examples of items they could add, including laptops, medications, food, extra clothing, toiletries, and so on.

### Seeking Emergency Care

#### When to Get Help

Caregivers reviewed the information contained in the *when to get help* section of the emergency action plan. Almost all caregivers (7/8, 88%) commented that the information presented in this section is clear. Specifically, they appreciated the capacity to call directly from the app if their child was experiencing a serious symptom. The addition that 13% (1/8) of the participants requested was for *uncontrolled pain* to be added as a reason to seek care.

#### When You Arrive

When asked to examine the information in the *when you arrive* section, all caregivers stated that the information would be helpful if they ever had to go to an ED outside of their treating institution. Caregivers stated that the information was very useful to explain general details about what the child is going through. A caregiver stated that defining what a fever is for a child with cancer would be helpful to add to the card, and another caregiver thought that port needle size was important to be included.

### Medical Management of Care

#### Logging Symptoms

Caregivers were asked to log a series of commonly monitored symptoms, which included fever, pain, poop, nausea and vomiting, and breathing. All caregivers were able to log symptoms successfully. Once a symptom was logged, they were asked if they wanted to continue tracking and at what time intervals they wanted to be reminded to check again. They also had the option to set medication reminders for certain medicines as needed. Caregivers were told by the interviewer that medications and dosages available for their symptoms would be entered by the medical team; however, this aspect was not available for this phase of testing. Then, the caregivers were asked to go through and track each symptom and were asked if tracking of each symptom was clear and easy to do. For example, caregivers said that it was easy to track headache, and they appreciated the different faces showing levels of pain or discomfort.

Some caregivers stated that a description under different nausea and vomiting faces would be helpful, as some symptoms such as nausea and vomiting and pooping had only a number identifier, unlike pain, which included a number scale and a descriptor of the level of pain. Of the 8 caregivers, 5 (63%) caregivers thought that the poop scale was not self-explanatory. A description of each type or definition of *normal* would make it easier to gauge.

All caregivers (8/8, 100%) said that tracking the temperature was clear and easy to do; however, 13% (1/8) of the caregivers was initially confused at the initiation of the emergency action plan when tracking a dangerously high temperature. Some comments were provided on the graphing ability of temperature symptom tracking to be able to view the tracking of multiple temperature readings over time.

#### Medications

Although medications were not available for review by the caregivers during alpha testing, the *give medications* option was shown during symptom tracking, and caregivers were asked about their thoughts on using the app for medication tracking and reminders. Overall, the caregivers were supportive of using the app for this purpose. However, 13% (1/8) of the caregivers expressed concern over whether they would trust the medication information contained within the app.

#### Completing a Symptom Tracking Event

Caregivers were asked how they would end a tracking event if they no longer wanted to track a symptom. Of the 8 caregivers, 5 (63%) caregivers did not have any problems in understanding how to end the tracking. The remaining 38% (3/8) of the caregivers needed to be guided through the process, and 33% (1/3) of them said that it was clear after they were shown what to do. Improved ease and clarity are needed in how to complete a tracking event.

### Miscellaneous App Functions

#### Pulsing Heart Perceptions

Caregivers were asked what they thought the *pulsing hearts* on the home screen meant after they tracked a symptom—more than one symptom at a time can have a *pulsing heart*. Most caregivers (7/8, 88%) knew that the heart meant that they were tracking that particular symptom. Only 13% (1/8) of the caregivers thought that the pulsing heart meant that the symptom being tracked was *good*.

#### To-do List Section

All reminders that caregivers have set show up in the *care tasks* section. Caregivers were asked if there was anything else that they would like to see in this section. Multiple caregivers mentioned that they would like to see medication (7/8, 88%) and appointment reminders (4/8, 50%) in this section.

#### Overall Impression

When asked if there were any final comments or concerns, several caregivers expressed their overall satisfaction with the app and could envision that it would serve a meaningful purpose to caregivers of children with cancer. Several key quotes from caregivers included the following:

I mean, it seems like you guys have everything on here right now that we’ve ran into.

I just want to tell you guys kudos because this is very self-explanatory. I feel like it’s very easy to follow and to understand...It’s user friendly, and I don’t feel intimidated by this program. I’m like oh, this is really awesome, and this makes good sense, and it walks me through if ever there’s a time where I’m questioning it pretty well, once I put it in there, it’s telling me yeah, you need to be calling the doctor.

It looks good. We’re kind of 30 weeks in, but at the beginning to have all that information available would be very helpful, for sure.

## Discussion

### Principal Findings

In this mixed methods study, we document the process and importance of involving key stakeholders in the prototyping and alpha testing of an mHealth app to support caregivers in the medical management of their child with cancer. The use of an app, such as Cope 360, that has been co-designed and created with input from the intended users has the potential to positively impact caregiver outcomes. Overall, the app was well accepted by caregivers of children with cancer, but several key issues arose that require refinement before further studies. Before we can explore the impact of this app, future work will need to be conducted to explore user experience and preferences in the real-world setting.

On the basis of the results of the qualitative exploration of the interviews with caregivers of children with cancer, several key refinements will be made to the Cope 360 app. We describe these based on two categories: medical information and terminology and more clarity in design and features to be included in the beta phase of testing. Then, we explain some next steps and the importance of human-centered design when designing mHealth apps to assist caregivers.

### Medical Information and Terminology

We found the importance of using proper, clear, and consistent terminology for medical terms. For example, the terminology regarding the type of line given to a particular patient was not universally well understood by caregivers, and the wording of the line type needed to change to *tunneled central line* instead of *central venous catheter*. We also learned about the importance of including more details concerning the patient’s medical information. For example, it was important to allow the addition of the size of the port needle to be part of the patient information in the app. On the *when you arrive* section to show to the emergency providers, caregivers also desired that the definition of fever be added, as this is a more complicated and specific definition than that used in general pediatrics [31].

### Improved Clarity in Design and Features to Be Included

Caregivers needed more clarity in the design of specific features to facilitate use in general and increase understanding for the first-time user. In the areas where the caregiver could add tasks or items to a list, there was confusion about the location and intent of the plus button; therefore, this was recommended to change to only show the plus button on the left-hand side of the screen with the option for typing content after the plus button was pressed. For symptom tracking, caregivers requested more consistency between the types of symptom tracking with the addition of descriptors along with numerical representation for nausea and vomiting. The Bristol poop scale was not easily understood, and caregivers desired an option for *no poop* when their child attempted but was unable to poop. Therefore, refinement to the poop scale was made with descriptors and an option for *no poop*. All symptom tracking charts were updated to include the date the symptom was tracked along with the time. These recommendations were relayed to the app development company.

### Future Steps and Directions

Very often, the intended end users are not included in the up-front design and creation of the mobile technology app [32-34]. This often results in the end product not aligning with the experiences and needs of the end user, which ultimately leads to poor uptake of the app or its incorporation into daily life. For this app, we leaned on the lived experience of caregivers of children with cancer to understand the intricacies of their experience with medical management of their child. This helped us identify important gaps in the mHealth app and quickly identify ways in which we could adjust small features to better accommodate the caregivers’ desires and needs. Our goal is that by creating an mHealth app with caregivers and for caregivers, we will be able to positively impact their experience with providing medical care to their child with cancer. The Van Houtven Caregiver Intervention Organizing Framework suggests that interventions to improve a caregiver’s clinical skills and knowledge, psychosocial (self-efficacy and coping) competency, support seeking (organizational and coordination), and quantity of caregiving will lead to benefits for both them and the patient with cancer [35]. For this project, we co-designed and created an app that is intended to improve caregivers’ clinical skills and knowledge, self-efficacy, and support seeking skills.

Although we were successful in collaborating directly with caregivers of children with cancer, our success was also dependent on input from our nurses who acted as proxy users. The nurses included in this study provide phone triage to caregivers of children with cancer regularly as part of their roles as nurse coordinator. They have the advantage of understanding the clinical context of the symptoms that we are tracking and the communication needs of the medical team when helping the caregiver relay questions or concerns. The disadvantage may have been that the nurses approach symptom tracking and emergency preparedness as a daily activity, whereas this can be a new and daunting task for caregivers. By combining the nurse coordinators’ inputs with testing by caregivers, we are hopeful that we can create a both practical and supportive app for the medical management of children with cancer.

The results from the alpha testing reported in this paper provide both encouraging signs that the app is likely to be useful and clear next steps for certain features and functionality. On the basis of these findings, we are planning a beta test with increased functionality and revised features and interface. Specific features that are anticipated to be explored further during beta testing include perceptions or alterations to the pulsing heart that signals an active symptom tracking, preferences for how to stop tracking an event, allotted time intervals for rechecking a symptom, and any additional recommendations for medical knowledge related to when to seek emergency care. Our intent is to use the mHealth app as part of a randomized controlled trial that specifically measures caregiver outcomes including mastery of caregiving, caregiver self-efficacy, and stress [36-39].

### Limitations

All the participants in this study, including proxy users (ie, nurse coordinators) and caregivers, were recruited from a single institution. There was a lack of male participants and those with race and ethnic backgrounds other than White, non-Hispanic. However, we received varying opinions on certain features and were able to collect data until no further themes emerged. Of note, alpha testing began when the COVID-19 pandemic changed the way in which we interacted with patients and research participants. The research team transitioned all recruitment, interview, and evaluation processes to a web-based platform. Although the interviews were audio recorded and transcribed, the opportunity to observe in more detail how the caregivers interacted with the app was hindered.

### Conclusions

By using a mixed methods approach for prototyping and alpha testing, we were able to create and refine an app to support caregivers in the medical management of their child with cancer. By placing the intended users at the forefront of the app design process, we created an app that was well received by caregivers of children with cancer. However, some key features require refinements based on collective feedback. Future research should focus on how caregivers can use this app in real life to manage the medical needs of their child with cancer. Refinements after real-life testing would allow large trials to evaluate the impact of this app on caregiver outcomes, such as caregiver’s feeling of self-efficacy and mastery of caregiving.

## Abbreviations

ED: emergency department
mHealth: mobile health
SUS: System Usability Scale
